# Prognosis of patients with prostate cancer and bone metastasis from the Japanese Prostatic Cancer Registry of Standard Hormonal and Chemotherapy Using Bone Scan Index cohort study

**DOI:** 10.1111/iju.14614

**Published:** 2021-06-19

**Authors:** Kenichi Nakajima, Atsushi Mizokami, Hideyasu Matsuyama, Tomohiko Ichikawa, Go Kaneko, Satoru Takahashi, Hiroaki Shiina, Hiroyuki Horikoshi, Katsuyoshi Hashine, Yutaka Sugiyama, Takeshi Miyao, Manabu Kamiyama, Kenichi Harada, Akito Ito, PROSTAT‐BSI Investigators

**Affiliations:** ^1^ Department of Functional Imaging and Artificial Intelligence Kanazawa University Kanazawa Japan; ^2^ Department of Integrative Cancer Therapy and Urology Kanazawa University Graduate School of Medical Science Kanazawa Japan; ^3^ Department of Urology Graduate School of Medicine Yamaguchi University Ube Japan; ^4^ Department of Urology Graduate School of Medicine Chiba University Chiba Japan; ^5^ Department of Uro‐Oncology Saitama Medical University International Medical Center Saitama Japan; ^6^ Department of Urology Nihon University School of Medicine Tokyo Japan; ^7^ Department of Urology Shimane University Faculty of Medicine Shimane Japan; ^8^ Department of Diagnostic Radiology Gunma Prefectural Cancer Center Ota Japan; ^9^ Department of Urology National Hospital Organization Shikoku Cancer Center Matsuyama Japan; ^10^ Department of Urology Graduate School of Medical Sciences Kumamoto University Kumamoto Japan; ^11^ Department of Urology Gunma University Graduate School of Medicine Maebashi Japan; ^12^ Department of Urology University of Yamanashi Yamanashi Japan; ^13^ Division of Urology, Department of Surgery Related Kobe University Graduate School of Medicine Kobe Japan; ^14^ Department of Urology Iwate Medical University Yahaba Japan

**Keywords:** Bone Scan Index, castration‐resistant prostate cancer, hormone‐sensitive prostate cancer, multicenter study, survival analysis

## Abstract

**Objective:**

To determine prognostic factors including the Bone Scan Index in prostate cancer patients receiving standard hormonal therapy and chemotherapy.

**Methods:**

This multicenter Prostatic Cancer Registry of Standard Hormonal and Chemotherapy Using Bone Scan Index study involved 30 hospitals and enrolled 247 patients (age 71 ± 8 years) with metastatic hormone‐sensitive prostate cancer (*n* = 148) under hormone therapy and metastatic castration‐resistant prostate cancer (*n* = 99) under chemotherapy. The Bone Scan Index (%) was determined by whole‐body bone scintigraphy using ^99m^Tc‐methylenediphosphonate. Patients were classified into tertiles and binary groups, and predictors of all‐cause death including Bone Scan Index, prostate‐specific antigen, and bone metabolic markers were determined using survival and proportional hazard analyses.

**Results:**

During a mean follow‐up period of 716 ± 404 days, 81 (33%) of the patients died, and 3‐year mortality rates were 20% and 52% in the metastatic hormone‐sensitive prostate cancer and metastatic castration‐resistant prostate cancer groups, respectively. Survival analysis showed that a Bone Scan Index >3.5% was a significant determinant of death in the metastatic hormone‐sensitive prostate cancer group, whereas prostate‐specific antigen >55 ng/mL before chemotherapy was a determinant of prognosis in the metastatic castration‐resistant prostate cancer group. A Bone Scan Index >3.5% was also associated with a high incidence of prostate‐specific antigen progression in the metastatic hormone‐sensitive prostate cancer group. Patients with metastatic hormone‐sensitive prostate cancer and a better Bone Scan Index response (>45%) to treatment had lower mortality rates than those without such response.

**Conclusion:**

The Bone Scan Index and hot spot number are significant determinants of 3‐year mortality, and combining the Bone Scan Index with prostate‐specific antigen should contribute to the management of prostate cancer patients with bone metastasis.

**Abbreviations & Acronyms**
1CTP: cross‐linked telopeptide parts of type I collagen
ADT: androgen deprivation therapy
ALP: alkaline phosphatase
ARTA: androgen receptor‐targeted agent
BAP: bone alkaline phosphatase
BMA: bone‐modifying agent
BSI: Bone Scan Index
CRP: C‐reactive protein
HSN: hot spot number
IFCC: International Federation of Clinical Chemistry and Laboratory Medicine
JSCC: Japan Society of Clinical Chemistry
mCRPC: metastatic castration‐resistant prostate cancer
mHSPC: metastatic hormone‐sensitive prostate cancer
NS: not significant
PROSTAT‐BSI: Prostatic Cancer Registry of Standard Hormonal and Chemotherapy Using Bone Scan Index
PSA: prostate‐specific antigen

## Introduction

Prostate cancer is the most frequently diagnosed type of cancer among men in developed countries, exceeding 1 million annually, and it is prevalent in Japan.^1^ The incidence of latent prostate cancer is also considered high, and this is recognized as a slow‐growing type.^2^ The advent of PSA might have enhanced the detectability of prostate cancer, but it remains a leading cause of death, particularly when associated with bone metastasis.^3^

The first‐line treatment for progressive prostate cancer is hormonal (ADT) or a combined androgen blockade, which has been widely applied to patients with metastatic prostate cancer.^4^ At a more progressive stage that is refractory to standard hormone therapy, or the phase of mCRPC, chemotherapy with docetaxel has commonly been administered.

As prostatic cancer frequently metastasizes to bone, early detection and a surrogate marker of the severity and therapeutic effects against bone metastasis is required. Although numbers of metastases and grades of diseases have been applied even in multicenter registries, such semiquantitative approaches generate crude parameters for accurately following up the amount of metastasis.^5^, ^6^ However, after the advent of BSI to quantify the total amount of bone metastasis, its diagnostic and prognostic roles, as well as clinical usefulness have been validated.^7^, ^8^, ^9^, ^10^, ^11^ As software for calculating BSI is installed in >800 hospitals in Japan, neural network‐based quantitation using BSI is a good background for evaluating patients with prostate cancer with bone metastasis nationwide.^12^, ^13^

In this context, we started the PROSTAT‐BSI study to evaluate standard hormonal therapy and chemotherapy incorporating BSI, and patients were recruited from 30 hospitals.^14^ The present study summarizes the findings of a nationwide prognostic study of the role of bone metastatic markers in standard hormonal therapy for mHSPC and chemotherapy for mCRPC.

## Methods

### Patients

Patients with bone metastasis (age 71 ± 8 years), who were scheduled to undergo hormonal therapy for prostate cancer with bone metastasis and chemotherapy (docetaxel) for the metastasis, were included in the study (Table 1). The patients were assessed by ^99m^Tc‐methylenediphosphonate bone scintigraphy, and at least one documented bone metastasis was confirmed by X‐ray computed tomography and/or magnetic resonance imaging. When a patient refractory to hormonal therapy was switched to chemotherapy, the patient was judged as censored alive and added to the chemotherapy group as a new patient (*n* = 27), and the markers just before the chemotherapy were used as a baseline condition. Finally, 148 and 99 patients were scheduled for hormonal therapy and chemotherapy, respectively.

**Table 1:** Characteristics of the patients

	Total	mHSPC	mCRPC	*P*
No. patients	247	148	99	
Age (years)	70.5 ± 7.8	71.0 ± 7.8	69.8 ± 7.9	0.23
Follow up (days)	716 ± 404	801 ± 403	591 ± 374	<0.0001
Events				
All‐cause death	81 (33%)	30 (20%)	51 (52%)	<0.0001†
Prostate cancer death	64 (26%)	22 (15%)	42 (42%)	<0.0001†
Relapse/progression	177 (72%)	92 (62%)	85 (86%)	<0.0001
Gleason score (median)	9	9	9	0.59†
Gleason score ≤7/8/9/10/unknown (%)	29/63/111/27/17 (11/26/45/11/7%)	14/40/65/17/12 (10/29/48/13/8%)	15/23/46/10/5 (15/23/47/10/5%)	
Non‐regional lymph node metastasis	31%	29%	32%	0.51†
Lung metastasis (%)	14%	14%	14%	0.99†
Liver metastasis (%)	2%	0.7%	4%	0.06†
Blood samples				
PSA (ng/mL)	781 ± 2308 (median 104)	1226 ± 2896 (median 261)	114 ± 220 (median 21)	<0.0001
ALP (IU/mL)‡	738 ± 1245	849 ± 1500	571 ± 684	0.09
BAP (μg/L)	67 ± 107	77 ± 124	52 ± 75	0.09
1CTP (ng/mL)	8.7 ± 9.2	8.6 ± 9.5	8.7 ± 8.7	0.93
Hemoglobin (g/dL)	12.7 ± 2.1	13.3 ± 2.2	11.9 ± 1.7	<0.0001
CRP (mg/dL)	1.5 ± 3.6	1.7 ± 4.1	1.3 ± 2.7	0.46
Bone scan at time of entry				
BSI (%)	3.2 ± 3.4	3.2 ± 3.6	3.4 ± 3.3	0.68
Probability of abnormality§	0.88 ± 0.20	0.85 ± 0.23	0.93 ± 0.15	0.007
Hot spots (*n*)	32 ± 36	30 ± 35	36 ± 37	0.19

† Pearson statistics.

‡ Measurement according to JSCC, which can be corrected to that of IFCC: ALP (IFCC = 0.35 × ALP [JSCC]).

§ Probability of abnormality was calculated by BONENAVI software indicating probability of metastases from whole‐body scan.

### Study design and end‐points

This multicenter observational study of patients with prostate cancer did not include specific therapeutic interventions or randomization. The primary end‐point was the prognosis of the patients after starting the hormonal therapy and chemotherapy, and it included all‐cause and prostate cancer death. The timing of progression or relapse after temporary improvement was determined by clinical progression including PSA and BSI determined every 3 months for 12 months of follow‐up. The total follow‐up time was 3 years.

### Bone scintigraphy and BSI

The patients were assessed by whole‐body bone scintigraphy before, and every 3 months for 12 months, then 2 and 3 years after treatment. The BSI and HSN were calculated using BONENAVI software (FUJIFILM Toyama Chemical, Tokyo, Japan).^13^ The rate (%) of BSI improvement was defined as a change from a higher BSI at 0 or 3 months to a lower value at 3 or 6 months, regardless of flare phenomenon.

### Bone metabolic markers and blood sampling

Metabolic bone markers included serum 1CTP, serum ALP and BAP. The blood parameters of hemoglobin and CRP were also assayed.

### Treatments

The patients were administered with standard hormonal therapy as part of an androgen blockade, and docetaxel chemotherapy. The administration and timing of bicalutamide, flutamide, abiraterone, enzalutamide and cabazitaxel were not regulated during the clinical course. The use of BMA including zoledronic acid and denosumab was not regulated.

### Ethics approval

The study protocols were approved by the Ethics Committee at the core center, Kanazawa University, and all the participating hospitals.

### Clinical trial registration

This multicenter study was registered on 1 May 2012 in the University Medical Information Network in Japan as UMIN00000‐7858.

### Statistical analysis

All values are shown as the mean ± standard deviation or as the median, as appropriate. Pairs of variables were compared using *t*‐tests and analyses of variance. Data without normal distribution were also assessed by non‐parametric analyses. Contingency tables were analyzed using Pearson statistics. The significance of univariable and multivariable analyses was determined using a proportional hazards model. Multivariable analysis with nominal logistic fit included selected variables, and the area under the receiver operating characteristics curve was calculated. The survival of the two groups of patients was analyzed using Cox models and log‐rank tests. Although binary and tertiary classification were initially applied, subsequent appropriate classification was added with reference to the initial results. Values with *P* < 0.05 were considered significant. Data were statistically analyzed using jmp version 14.3 (SAS Institute, Cary, NC, USA).

## Results

### Patient demographics

Gleason scores, non‐regional lymph node and distant organ metastases did not significantly differ between patients with mCRPC and mHSPC. The PSA value was higher in the mHSPC than the mCRPC group. Among the mHSPC group, 123 (83%) were administered with a combined androgen blockade (standard androgen deprivation plus bicalutamide) as first‐line hormonal therapy. When patients relapsed after standard hormonal therapy, second‐line or subsequent therapy was administered to 69 (47%) patients, with flutamide in 37 (25%), ARTA (enzalutamide and abiraterone + prednisolone) in 39 (26.4%) and docetaxel in 25 (17%). Eight (8%) patients with mCRPC were administered with ARTA before docetaxel, and 48 (48%) and 15 (15%), respectively, were treated with ARTA and cabazitaxel after progression.

### Outcome events

During a mean follow‐up period of 716 ± 404 days, 81 (33%) of 247 patients died from any causes, and 64 (26%) died from prostate cancer. The disease progression was judged by PSA in 177 (72%) of the patients. Mortality rates (%) and progression were significantly higher in patients with mCRPC than mHSPC (Table 1).

### BSI versus number of hot spots

**Fig. 1:**
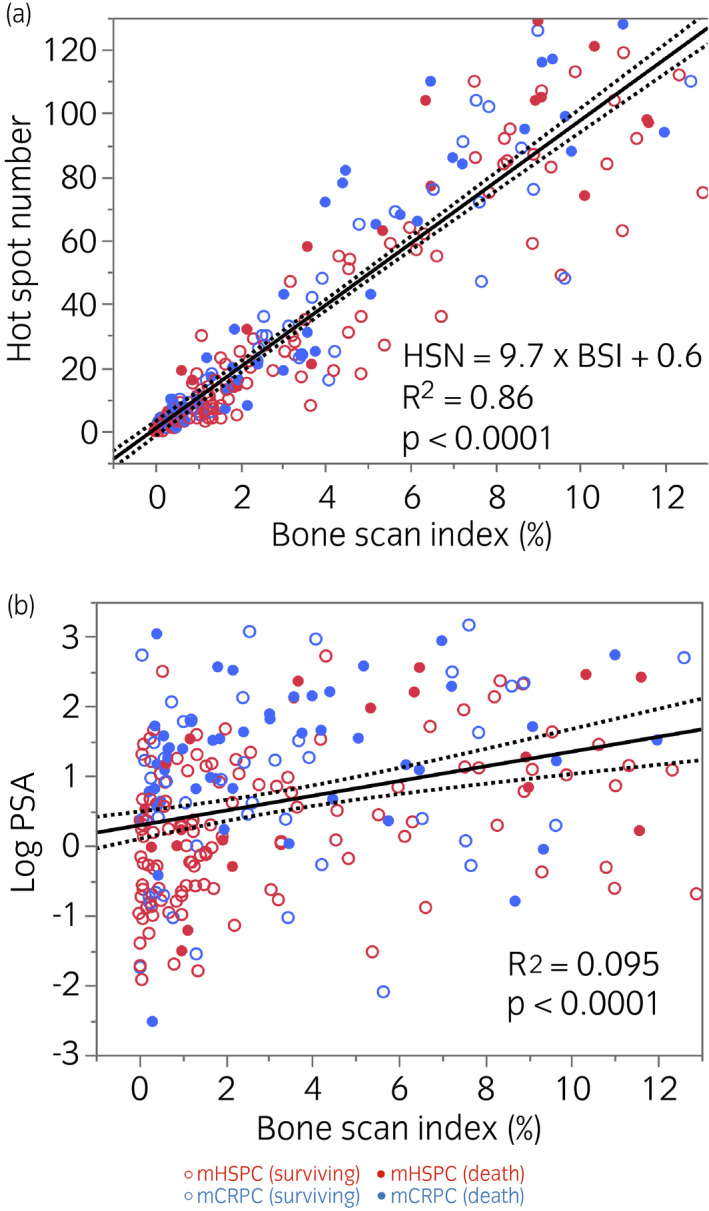
Linear regression line and confidence ranges (dotted line) of hot spots, PSA and BSI. (a) HSN and BSI (%), and (b) PSA (ng/mL; log scale) versus BSI (%).

The average BSI and number of hot spots were comparable between the mHSPC and mCRPC groups (Table 1). The number of hot spots linearly correlated with BSI (%) (HSN = 9.7 × BSI − 0.6, *R* ^2^ = 0.86, *P* < 0.0001); however, the correlation between PSA level and BSI was relatively weak (*R* ^2^ = 0.095; Fig. 1). The BSI flare phenomenon occurred in 12% and 28% of the patients with mHSPC and mCRPC, respectively (*P* = 0.0082).

### Survival analysis

**Fig. 2:**
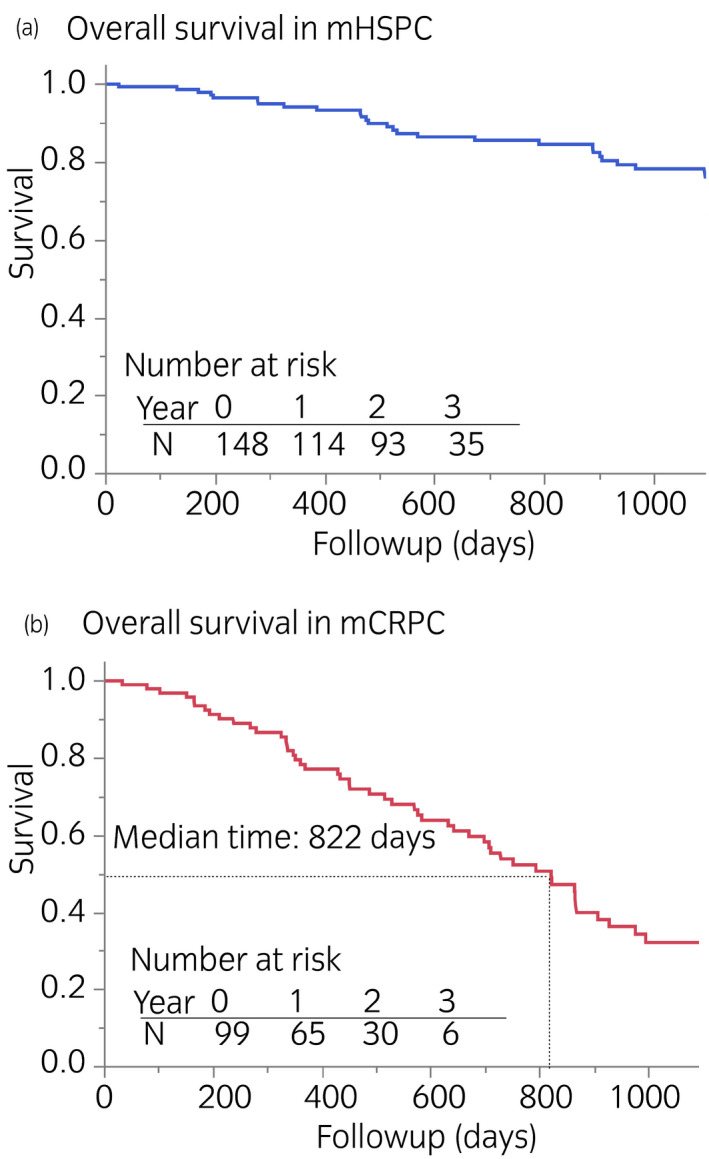
Survival analysis in patients with (a) mHSPC and (b) mCRPC.

**Fig. 3:**
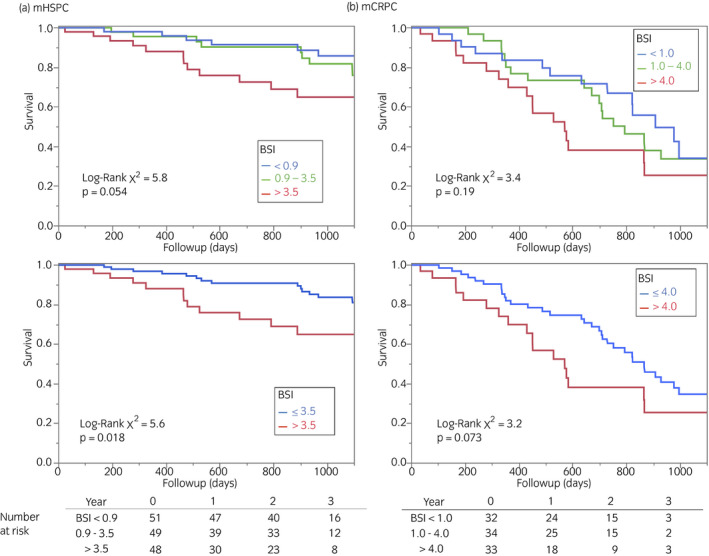
Survival of patients according to tertiles and binary subgroups. Upper panels, tertiles of BSI. Lower panels, binary high versus low BSI according to cut‐off 3.5 and 4.0 in patients with (a) mHSPC and (b) mCRPC, respectively.

**Fig. 4:**
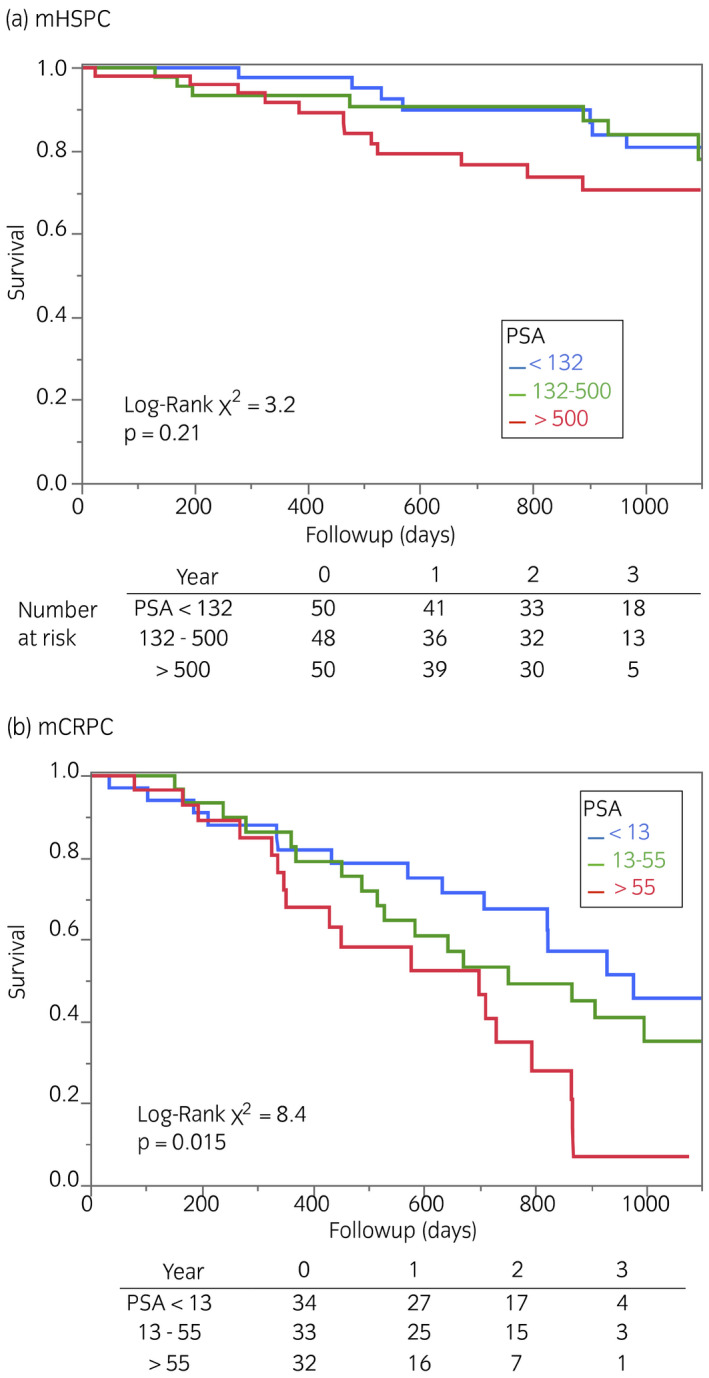
Survival analysis of (a) mHSPC and (b) mCRPC tertiles of PSA values. PSA (ng/mL).

We analyzed the survival of the patients with mHSPC and mCRPC (Fig. 2). During 3 years of follow up, all‐cause deaths accounted for 20% and 52% in the mHSPC and mCRPC groups, respectively (*P* < 0.0001). The presence or absence of visceral (lung or liver) metastases did not contribute to the overall survival of the mHSPC group during the follow‐up period. When the patients were equally classified into tertiles based on BSI, the cut‐off values were <0.9%, 0.9–3.5% and >3.5% for the mHSPC group, and <1%, 1–4% and >4% for the mCRPC group. Survival differed only in the mHSPC group with a borderline significance (*P* = 0.054), and groups with BSI of <0.9 and 0.9–3.5 did not significantly differ (Fig. 3). When the patients with mHSPC were classified according to a high or low BSI using a threshold of 3.5, mortality was significantly higher in groups with high BSI than low BSI (*P* = 0.018). In contrast, mortality tended to higher among patients with mCRPC and high BSI, but the difference did not reach significance, even with binary classification (*P* = 0.073). Likewise, PSA tertiles of patients showed that the initial PSA level was a determinant of prognoses in patients with mCRPC (*P* = 0.015), but not mHSPC (*P* = 0.21; Fig. 4).

### Survival of PSA and BSI progression

**Fig. 5:**
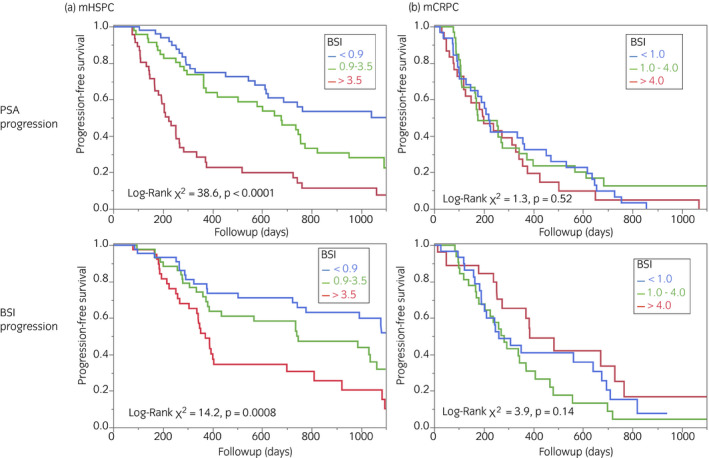
Progression‐free survival of PSA and BSI in (a) mHSPC and (b) mCRPC tertiles based on BSI.

Relationships between time to PSA progression and BSI tertiles were evaluated (Fig. 5). The prognosis was significantly worse in the mHSPC group with a higher BSI (*P* < 0.0001), but did not differ in the mCRPC group. Survival until the start of BSI progression was worse among patients in the higher BSI tertiles (*P* = 0.0008) for mHSPC, but did not significantly differ in the mCRPC group.

### Death predicted by proportional hazard analysis

All‐cause death among patients in the mHSPC group was assessed by univariate proportional hazards analysis (Table 2). Among the variables, BAP, 1CTP, CRP, hemoglobin, BSI and number of hot spots were significantly associated with death. However, multivariate proportional hazards analysis selected only the number of hot spots (*P* = 0.037) in the mHSPC group when adjusted for other variables. The same variables were associated with death among patients with mCRPC (Table 3), whereas multivariate proportional hazards analysis found that none of the variables were significant, and CRP was borderline significant (*P* = 0.087). When the contribution of PSA and the BSI to all‐cause death was compared, BSI was more significant (*P* = 0.011) than PSA (*P* = NS) in the mHSPC group, but neither was significant in the mCRPC group (*P* = 0.061 and NS for BSI and PSA, respectively; Tables 2,3).

**Table 2:** Proportional hazards and multivariable analysis of PSA and BSI in patients with mHSPC

Term	Hazard ratio	Lower 95%	Upper 95%	Wald χ^2^	*P*
Proportional hazards analysis					
Gleason score	1.064	0.779	1.568	0.121	0.73
Gleason score ≥9	1.931	0.844	4.418	2.432	0.12
Liver/lung metastasis	0.943	0.329	2.707	0.019	0.91
PSA (ng/mL)	1.00002	0.9999	1.0001	0.184	0.67
ALP (IU/mL)	1.0001	0.9999	1.0002	2.705	0.10
BAP (μg/L)	1.004	1.001	1.006	10.262	0.0014
1CTP (ng/mL)	1.054	1.016	1.086	10.680	0.0011
CRP (mg/dL)	1.069	0.998	1.125	5.093	0.024
Hemoglobin (g/dL)	0.823	0.706	0.971	5.767	0.016
BSI (%)	1.125	1.023	1.229	6.451	0.011
BSI >3.5%	2.338	1.131	4.832	5.260	0.022
HSN	1.016	1.007	1.024	12.788	0.0004
Multivariable proportional hazards analysis of PSA and BSI					
PSA (ng/mL)	0.99996	0.99980	1.00001	0.326	0.57
BSI (%)	1.140	1.027	1.260	6.567	0.010

**Table 3:** Proportional hazards and multivariable analysis of PSA and BSI in patients with mCRPC

Term	Hazard ratio	Lower 95%	Upper 95%	Wald χ^2^	*P*
Proportional hazards analysis					
Gleason score	1.145	0.910	1.510	1.108	0.29
Gleason score ≥9	1.069	0.595	1.922	0.050	0.82
Liver/lung metastasis	1.543	0.749	3.180	1.386	0.24
PSA (ng/mL)	1.001	0.999	1.002	1.227	0.27
ALP (IU/mL)	1.001	1.000	1.001	20.495	<0.0001
BAP (μg/L)	1.006	1.003	1.010	13.778	0.0002
1CTP (ng/mL)	1.052	1.023	1.076	16.300	<0.0001
CRP (mg/dL)	1.203	1.068	1.332	10.306	0.0008
Hemoglobin (g/dL)	0.683	0.565	0.830	15.272	<0.0001
BSI (%)	1.102	1.005	1.220	4.664	0.031
BSI >4.0%	1.722	0.962	3.082	3.352	0.067
HSN	1.009	1.001	1.017	5.462	0.019
Multivariable proportional hazards analysis of PSA and BSI					
PSA (ng/mL)	1.0003	0.9987	1.0015	0.145	0.70
BSI (%)	1.095	0.993	1.201	3.524	0.061

### Effect of initial response rate in BSI on survival analysis

**Fig. 6:**
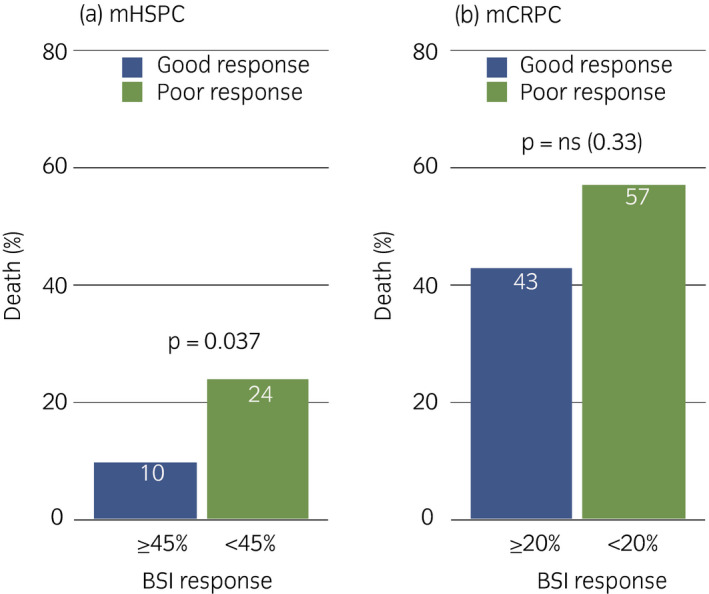
Comparison of good and poor responses using thresholds of 45% and 20% for (a) mHSPC and (b) mCRPC, respectively.

We divided the patients equally into two subgroups based on median BSI response rates (%) at 6 months after therapy. The thresholds were 45% and 20% for the mHSPC and mCRPC subgroup. Contingency analysis showed higher mortality rates for the group with a poor BSI response (*P* = 0.037) in the mHSPC group, whereas patients with mCRPC and good and poor BSI responses did not significantly differ (*P* = 0.33; Fig. 6).

### Effect of BMA

The BMA was used in 87 (59%) and 76 (77%) patients with mHSPC and mCRPC, respectively. The contingency table analysis showed that the use of BMA and mortality was not significantly correlated. Death occurred in 21% and 20% with and without BMA, respectively, in the mHSPC group (*P* = 0.88), and 54% and 43%, respectively, in the mCRPC group (*P* = 0.38).

## Discussion

The PROSTAT‐BSI was the first nationwide, prospective observational study of Japanese patients with prostate cancer who were treated with standard hormonal therapy and docetaxel chemotherapy.^14^ The 3‐year follow‐up results showed that a BSI of >3.5% was a significant determinant of all‐cause death in patients with mHSPC. Although multiple factors were involved in the prognosis of mCRPC with bone metastasis, CRP tended to be higher in patients with a poor prognosis according to multivariate analyses.

During the diagnostic process of prostate cancer, after initial medical checkup in clinics, serum PSA is measured as the key to the first workup, followed by various imaging modalities, such as X‐ray computed tomography and magnetic resonance imaging, depending on the patient situation. A definitive diagnosis is usually based on prostate biopsy specimens, and subsequent treatment strategies depend on consideration of Gleason scores, PSA levels, clinical stage and metastatic locations.^15^, ^16^

Although PSA is measured in routine clinical practice, limitations for patients with mCRPC have been recognized due to the androgen‐dependent nature of PSA.^17^ The levels of PSA during tumor regression in mHSPC might decrease in cancer cells due to inactivation of the PSA promoter by androgen deprivation, whereas they might not necessarily parallel the amount of bone metastasis in mCRPC.^14^

Bone scintigraphy remains the first‐line whole‐body survey of metastasis, even after the advent of X‐ray computed tomography and magnetic resonance imaging. Although hot spots have been counted in previous prostate cancer working group activities,^5^, ^6^ recent advances in quantitation using artificial intelligence have enhanced their diagnostic utility for initial diagnosis and follow‐up studies.^18^, ^19^

The National Comprehensive Cancer Network recommended a bone scan at the time of onset and every 6–12 and 8–12 weeks for ADT and CRPC, respectively (version 2.2021, https://www.nccn.org/professionals/physician_gls/pdf/prostate.pdf). Although we carried out bone scans every 3 months in the present study, we agree with this recommendation in clinical practice. However, although a bone scan at 3 months might reflect flare phenomenon, subsequent therapeutic response could be assessed by the follow‐up studies using the initial response. We therefore recommend a bone scan every 3–6 months just after the start of the treatment and significant changes in PSA, depending on the patient conditions.

The prognosis is poor for patients with an initial BSI >3.5% who are indicated for standard hormonal therapy, as this value usually indicates a large number of bone metastases. However, we found that overall survival did not significantly differ between patients with BSI <0.9% and those with BSI 0.9–3.5%. A European study of patients with ADT significantly associated BSI with overall survival; BSI 0%, ≤1% and >1% stratified patient prognosis from good to poor.^9^ Another study of high‐risk prostate cancer patients under primary hormonal therapy found that the 5‐year mortality among patients with metastasis was significantly high in high‐BSI patients.^8^ Thus, although the thresholds for subgroups might differ depending on the patient backgrounds and study protocols, all studies to date have supported the relevance of the BSI to survival.

A retrospective analysis of patients with mCRPC found that a change in BSI while adjusting for PSA was prognostic at 3 and 6 months of treatment, whereas PSA was not prognostic when adjusted for BSI.^20^ A recent large‐scale clinical trial of tasquinimod involving patients with mCRPC also associated the higher BSI with poorer overall survival.^19^ Although the tendency was similar in the tertile analysis in the PROSTAT‐BSI study, the difference between groups with high and low BSI did not reach statistical significance (*P* = 0.05–0.10).

However, considering contemporary second‐line and subsequent therapies, such as flutamide, abiraterone, enzalutamide and docetaxel, in patients with mHSPC in the present study, even when cancer recurred under the first‐line hormonal therapy, survival might be extended if BSI is <3.5%.^21^, ^22^, ^23^, ^24^ In contrast, in patients with mCRPC, although univariable analysis associated multiple factors in outcomes, multivariable analysis did not identify a single variable that critically impacted prognosis. By this stage, serum PSA level, bone destruction and osteoblastic activity, anemia, and inflammatory response (CRP), and an increased incidence of organ metastasis could synergically affect general deterioration.

Several background characteristics in PROSTAT‐BSI need to be considered. Although the effect of the Gleason score was not statistically high in the present study, it might have been caused by the high proportion (87%) of patients with scores ≥8. As the prognosis of patients with BSI ≤3.5% did not significantly differ, this might be explained partly by the better responses of Japanese patients to standard hormonal and subsequent therapies compared with white men, as clinical outcomes after hormonal therapy are quite different between Japanese American and white American men.^25^ In addition, metastasis to the lungs and liver did not affect overall survival in the mHSPC group, which might have been partly due to the small amount of lung metastasis or the possibility of effective responses to hormonal and chemotherapy among patients with lung metastasis. Another reason might be that few patients had lung and liver metastasis in the present study population.

All patients with mHSPC had responded to hormonal treatment to some degree by 6 months after therapy; just 10% of those with a favorable response of ≥45% died within 3 years of follow up. In contrast, the death rate of those with a relatively poor BSI response of <45%, was 2.4‐fold higher, and the rate of BSI progression was higher. The progression of PSA and BSI was equally high in both good and poor BSI response groups. Even when docetaxel somewhat improved bone metastases, it might not have improved the prognosis of the mCRPC group, because they had mixed status, such as worsening systemic conditions, anemia, inflammatory responses and visceral metastases. Others have also found that the assessment of response rates and the BSI has prognostic significance, and the tendency was similar in this Japanese cohort with mHSPC.^20^, ^26^, ^27^ Although the amount of bone metastasis at baseline plays a critical role in prognosis, the response rate just after treatment could stratify patient prognosis in conjunction with the baseline BSI, and the merit of quantitative evaluation, such as BSI, should be emphasized.

The follow‐up period of 3 years was relatively short for patients with mHSPC, considering that the prognosis of patients with prostate cancer in Japan is generally better than that in Europe and North America. One study found that the median survival of 5618 Japanese patients with metastatic mHSPC was ˜6 years.^28^ As the application of treatment options, such as bicalutamide, flutamide, abiraterone, enzalutamide, docetaxel and cabazitaxel, was not regulated in the present study, further investigation is required to determine their effects.

In conclusion, the PROSTAT‐BSI study showed that a BSI of >3.5% at the start of hormone therapy was a determinant of poor prognosis, whereas a BSI ≤3.5% was not. Multiple composite factors, such as PSA, BSI and CRP, could determine the prognosis of mCRPC. The integration of BSI and HSN into a conventional bone marker can assist the evaluations of therapeutic effects and the risk stratification of patients with metastatic prostate cancer.

## Conflict of interest

KN and AM have collaborative research works with FUJIFILM Toyama Chemical, Tokyo, Japan.

## References

[1] Torre LA, Bray F, Siegel RL*et al*. Global cancer statistics, 2012. CA Cancer J. Clin. 2015; 65: 87–108. 25651787 10.3322/caac.21262

[2] Konety BR, Bird VY, Deorah S, Dahmoush L. Comparison of the incidence of latent prostate cancer detected at autopsy before and after the prostate specific antigen era. J. Urol. 2005; 174: 1785–8. 16217287 10.1097/01.ju.0000177470.84735.55

[3] Fujimoto H, Nakanishi H, Miki T*et al*. Oncological outcomes of the prostate cancer patients registered in 2004: report from the Cancer Registration Committee of the JUA. Int. J. Urol. 2011; 18: 876–81. 22142466 10.1111/j.1442-2042.2011.02895.x

[4] Labrie F, Belanger A, Simard J, Labrie C, Dupont A. Combination therapy for prostate cancer. Endocrine and biologic basis of its choice as new standard first‐line therapy. Cancer 1993; 71: 1059–67. 8428328 10.1002/1097-0142(19930201)71:3+<1059::aid-cncr2820711426>3.0.co;2-6

[5] Scher HI, Halabi S, Tannock I*et al*. Design and end points of clinical trials for patients with progressive prostate cancer and castrate levels of testosterone: recommendations of the Prostate Cancer Clinical Trials Working Group. J. Clin. Oncol. 2008; 26: 1148–59. 18309951 10.1200/JCO.2007.12.4487 PMC4010133

[6] Scher HI, Morris MJ, Stadler WM*et al*. Trial design and objectives for castration‐resistant prostate cancer: updated recommendations from the prostate cancer clinical trials working group 3. J. Clin. Oncol. 2016; 34: 1402–18. 26903579 10.1200/JCO.2015.64.2702 PMC4872347

[7] Imbriaco M, Larson SM, Yeung HW*et al*. A new parameter for measuring metastatic bone involvement by prostate cancer: the Bone Scan Index. Clin. Cancer Res. 1998; 4: 1765–2. 9676853

[8] Kaboteh R, Damber JE, Gjertsson P*et al*. Bone Scan Index: a prognostic imaging biomarker for high‐risk prostate cancer patients receiving primary hormonal therapy. EJNMMI Res. 2013; 3: 9. 23384286 10.1186/2191-219X-3-9 PMC3570487

[9] Reza M, Bjartell A, Ohlsson M*et al*. Bone Scan Index as a prognostic imaging biomarker during androgen deprivation therapy. EJNMMI Res. 2014; 4: 58. 25386390 10.1186/s13550-014-0058-y PMC4205473

[10] Nakajima K, Edenbrandt L, Mizokami A. Bone Scan Index: a new biomarker of bone metastasis in patients with prostate cancer. Int. J. Urol. 2017; 24: 668–73. 28556293 10.1111/iju.13386

[11] Haupt F, Berding G, Namazian A*et al*. Expert system for bone scan interpretation improves progression assessment in bone metastatic prostate cancer. Adv. Ther. 2017; 34: 986–94. 28265811 10.1007/s12325-017-0505-z

[12] Horikoshi H, Kikuchi A, Onoguchi M, Sjostrand K, Edenbrandt L. Computer‐aided diagnosis system for bone scintigrams from Japanese patients: importance of training database. Ann Nucl. Med. 2012; 26: 622–6. 22729550 10.1007/s12149-012-0620-5 PMC3475966

[13] Nakajima K, Nakajima Y, Horikoshi H*et al*. Enhanced diagnostic accuracy for quantitative bone scan using an artificial neural network system: a Japanese multi‐center database project. EJNMMI Res. 2013; 3: 83. 24369784 10.1186/2191-219X-3-83 PMC3877947

[14] Nakajima K, Kaneko G, Takahashi S*et al*. Role of Bone Scan Index in the prognosis and effects of therapy on prostate cancer with bone metastasis: study design and rationale for the multicenter Prostatic Cancer Registry of Standard Hormonal and Chemotherapy Using Bone Scan Index (PROSTAT‐BSI) study. Int. J. Urol. 2018; 25: 492–9. 29633398 10.1111/iju.13556

[15] van den Bergh RC, Roemeling S, Roobol MJ*et al*. Prospective validation of active surveillance in prostate cancer: the PRIAS study. Eur. Urol. 2007; 52: 1560–3. 17532115 10.1016/j.eururo.2007.05.011

[16] Kakehi Y, Kamoto T, Shiraishi T*et al*. Prospective evaluation of selection criteria for active surveillance in Japanese patients with stage T1cN0M0 prostate cancer. Jpn. J. Clin. Oncol. 2008; 38: 122–8. 18272471 10.1093/jjco/hym161

[17] Mizokami A, Izumi K, Konaka H*et al*. Understanding prostate‐specific antigen dynamics in monitoring metastatic castration‐resistant prostate cancer: implications for clinical practice. Asian J. Androl. 2017; 19: 143–8. 27270339 10.4103/1008-682X.179159 PMC5312209

[18] Van den Wyngaert T, Strobel K, Kampen WU*et al*. The EANM practice guidelines for bone scintigraphy. Eur. J. Nucl. Med. Mol. Imaging 2016; 43: 1723–38. 27262701 10.1007/s00259-016-3415-4 PMC4932135

[19] Armstrong AJ, Anand A, Edenbrandt L*et al*. Phase 3 assessment of the automated Bone Scan Index as a prognostic imaging biomarker of overall survival in men with metastatic castration‐resistant prostate cancer: a secondary analysis of a randomized clinical trial. JAMA Oncol. 2018; 4: 944–51. 29799999 10.1001/jamaoncol.2018.1093 PMC6145727

[20] Dennis ER, Jia X, Mezheritskiy IS*et al*. Bone Scan Index: a quantitative treatment response biomarker for castration‐resistant metastatic prostate cancer. J. Clin. Oncol. 2012; 30: 519–24. 22231045 10.1200/JCO.2011.36.5791 PMC3295554

[21] Gillessen S, Omlin A, Attard G*et al*. Management of patients with advanced prostate cancer: recommendations of the St Gallen Advanced Prostate Cancer Consensus Conference (APCCC) 2015. Ann. Oncol. 2015; 26: 1589–604. 26041764 10.1093/annonc/mdv257 PMC4511225

[22] Reza M, Ohlsson M, Kaboteh R*et al*. Bone Scan Index as an imaging biomarker in metastatic castration‐resistant prostate cancer: a multicentre study based on patients treated with abiraterone acetate (Zytiga) in clinical practice. Eur. Urol. Focus 2016; 2: 540–6. 28723520 10.1016/j.euf.2016.02.013

[23] Davis ID, Martin AJ, Stockler MR*et al*. Enzalutamide with standard first‐line therapy in metastatic prostate cancer. N. Engl. J. Med. 2019; 381: 121–31. 31157964 10.1056/NEJMoa1903835

[24] Anand A, Morris MJ, Larson SM*et al*. Automated Bone Scan Index as a quantitative imaging biomarker in metastatic castration‐resistant prostate cancer patients being treated with enzalutamide. EJNMMI Res. 2016; 6: 23. 26960325 10.1186/s13550-016-0173-z PMC4785173

[25] Fukagai T, Namiki TS, Carlile RG, Yoshida H, Namiki M. Comparison of the clinical outcome after hormonal therapy for prostate cancer between Japanese and Caucasian men. BJU Int. 2006; 97: 1190–3. 16686710 10.1111/j.1464-410X.2006.06201.x

[26] Kaboteh R, Gjertsson P, Leek H*et al*. Progression of bone metastases in patients with prostate cancer – automated detection of new lesions and calculation of Bone Scan Index. EJNMMI Res. 2013; 3: 64. 23947784 10.1186/2191-219X-3-64 PMC3751570

[27] Reza M, Jones R, Aspegren J*et al*. Bone Scan Index and progression‐free survival data for progressive metastatic castration‐resistant prostate cancer patients who received ODM‐201 in the ARADES multicentre study. Eur. Urol. Focus 2016; 2: 547–52. 28723521 10.1016/j.euf.2016.01.005

[28] Kadono Y, Nohara T, Ueno S*et al*. Validation of TNM classification for metastatic prostatic cancer treated using primary androgen deprivation therapy. World J. Urol. 2016; 34: 261–7. 26047654 10.1007/s00345-015-1607-3

